# Idiopathic and normal lateral lumbar curves: muscle effects interpreted by 12th rib length asymmetry with pathomechanic implications for lumbar idiopathic scoliosis

**DOI:** 10.1186/s13013-016-0093-8

**Published:** 2016-10-14

**Authors:** Theodoros B. Grivas, R. Geoffrey Burwell, Vasileios Kechagias, Christina Mazioti, Apostolos Fountas, Dimitra Kolovou, Evangelos Christodoulou

**Affiliations:** 1 Department of Orthopaedics and Traumatology, “Tzaneio” General Hospital, Tzani and Afendouli 1, Piraeus, 18536 Greece; 2 Centre for Spinal Studies and Surgery, Nottingham University Hospitals NHS Trust, Queen’s Medical Centre Campus, Nottingham, UK; 3 Department of Radiology, “Tzaneio” General Hospital, Tzani and Afendouli 1 st, Piraeus, 18536 Greece; 4 Asklepios Klinik Lindenlohe, Schwandorf, Germany

## Abstract

**Background:**

The historical view of scoliosis as a primary rotation deformity led to debate about the pathomechanic role of paravertebral muscles; particularly multifidus, thought by some to be scoliogenic, counteracting, uncertain, or unimportant. Here, we address lateral lumbar curves (LLC) and suggest a pathomechanic role for quadrates lumborum, (QL) in the light of a new finding, namely of 12th rib bilateral length asymmetry associated with idiopathic and small non-scoliosis LLC.

**Methods:**

Group 1: The postero-anterior spinal radiographs of 14 children (girls 9, boys 5) aged 9–18, median age 13 years, with right lumbar idiopathic scoliosis (IS) and right LLC less that 10°, were studied. The mean Cobb angle was 12° (range 5–22°). Group 2: In 28 children (girls 17, boys 11) with straight spines, postero-anterior spinal radiographs were evaluated similarly to the children with the LLC, aged 8–17, median age 13 years. The ratio of the right/left 12th rib lengths and it’s reliability was calculated. The difference of the ratio between the two groups was tested; and the correlation between the ratio and the Cobb angle estimated. Statistical analysis was done using the SPSS package.

**Results:**

The ratio’s reliability study showed intra-observer +/−0,036 and the inter-observer error +/−0,042 respectively in terms of 95 % confidence limit of the error of measurements. The 12th rib was longer on the side of the curve convexity in 12 children with LLC and equal in two patients with lumbar scoliosis. The 12th rib ratios of the children with lumbar curve were statistically significantly greater than in those with straight spines. The correlation of the 12th rib ratio with Cobb angle was statistically significant. The 12th thoracic vertebrae show no axial rotation (or minimal) in the LLC and no rotation in the straight spine group.

**Conclusions:**

It is not possible, at present, to determine whether the 12th convex rib lengthening is congenitally lengthened, induced mechanically, or both. Several small muscles are attached to the 12th ribs. We focus attention here on the largest of these muscles namely, QL. It has attachments to the pelvis, 12th ribs and transverse processes of lumbar vertebrae as origins and as insertions. Given increased muscle activity on the lumbar curve convexity and similar to the interpretations of earlier workers outlined above, we suggest two hypotheses, relatively increased activity of the right QL muscle causes the LLCs (first hypothesis); or counteracts the lumbar curvature as part of the body’s attempt to compensate for the curvature (second hypothesis). These hypotheses may be tested by electrical stimulation studies of QL muscles in subjects with lumbar IS by revealing respectively curve worsening or correction. We suggest that one mechanism leading to relatively increased length of the right 12 ribs is mechanotransduction in accordance with Wolff’s and Pauwels Laws.

## Background

The historical view of scoliosis as a primary rotation deformity [1] led to debate about the pathomechanic role of paravertebral muscles; particularly multifidus, thought by some to be scoliogenic [2–4], counteracting [5, 6], uncertain [7], or unimportant [8]. Here, we address LLC and suggest a pathomechanic role for quadrates lumborum, (QL) in the light of a new finding, namely of 12th rib bilateral length asymmetry associated with idiopathic and small non-scoliosis LLC.

## Methods

### Ethical issues

Written consent was obtained from the patients or their relatives for publication of this study. IRB approval has been obtained, (21-5-2015 min of 42nd meeting of the “Tzaneio” General Hospital Scientific Council), for the implementation of this research.

### Definitions

We define Lateral Lumbar Curve (LLC) in the text as all the lumbar curves with a Cobb angle less than 10°. When LLC is 10° or more it is a scoliosis. The children with LLC less than 10° of Cobb angle are not characterized as having scoliosis.

### The examinees

We include subjects with scoliosis having LLC in our data base during the last 5 years, when this Scoliosis Outpatient Department (UPD) was started upon the appointment in the hospital of the first author. The older patients in the samples were included as part of all the registered children and adolescents with LLC in our scoliosis UPD.

A right LLC deemed of primary interest because it was our observation that in LLC the 12th pair of ribs did not have equal length. We included right lumbar only because the number of children with left lumbar curves we have in our registry is very small to analyse.

Two groups of children were formed. The postero-anterior (P-A) spinal radiographs of these two groups of examinees were assessed.

- **Group 1**: The P-A spinal radiographs of 14 children (girls 9, boys 5) with right lumbar idiopathic scoliosis (IS) and right LLC less that 10°, were studied, aged 9–18, median age 13 years. The mean Cobb angle of all these children was 12° (range 5–22°), Table 1. No neurological signs were found on clinical examination in all children suffering lumbar idiopathic scoliosis. The group 1 is smaller than group 2 because both the history of our scoliosis UPD is short and the incidence of LLC is very small as well.

**Table 1:** Details for children in group one

Number	Male(M) Female (F)	Age in years	Right 12th rib length in cm	Left 12th rib length in cm	ratio right/left 12th rib lengths	ratio left/right 12th rib lengths	Curve	Lumbar Cobb angle
1	F	18	7,5	7,2	1,04	0,96	RIGHT T11-L4 = 5	5
2	F	13	10,7	10,6	1,01	0,99	RIGHT T11-L4 = 5	5
3	M	10	6,8	6,8	1,00	1,00	RIGHT L1-L4 = 6	6
4	F	12	9,5	9,4	1,01	0,99	RIGHT T11-L4 = 8	8
5	F	10	7	6,8	1,03	0,97	RIGHT T12-L3 = 9	9
6	F	14	10,5	10,5	1,00	1,00	RIGHT T12-L4 = 9	9
7	M	9	7,1	6	1,18	0,85	RIGHT T12-L4 = 10	10
8	M	11	8,4	8	1,05	0,95	RIGHT L1-L4 = 13	13
9	M	15	6,2	5,6	1,11	0,90	RIGHT T10-L3 = 15	15
10	F	13	8,1	7	1,16	0,86	RIGHT T10-L3 = 15	15
11	F	13	7,4	6,8	1,09	0,92	RIGHT T12-L4 = 15	15
12	M	15	6,2	5,8	1,07	0,94	RIGHT T10-L4 = 19	17
13	F	12	9,4	8,8	1,07	0,94	RIGHT T7-L2 = 21	18
14	F	15	10,6	9,4	1,13	0,89	RIGHT T12-L4 = 22	22
- **Group 2**: In 28 children (girls 17, boys 11) with straight spines, the P-A spinal radiographs were evaluated similarly to the children with the LLC, aged 8–17, median age 13 years, Table 2.

**Table 2:** Details for children in group two

Number	Male(M) Female (F)	Age in years	Right 12th rib length in cm	Left 12th rib length in cm	ratio right/left 12th rib lengths	ratio left/right 12th rib lengths	Lumbar spine
1	F	9	8,7	9,1	0,96	1,05	STRAIGHT
2	F	14	10,5	10,8	0,97	1,03	STRAIGHT
3	M	12	10,7	10,7	1,00	1,00	STRAIGHT
4	M	12	8,9	9,5	0,94	1,07	STRAIGHT
5	F	12	10	10,4	0,96	1,04	STRAIGHT
6	M	12	9,1	9	1,01	0,99	STRAIGHT
7	F	12	3,7	4,1	0,90	1,11	STRAIGHT
8	F	13	10,6	11	0,96	1,04	STRAIGHT
9	F	13	4,2	4,4	0,95	1,05	STRAIGHT
10	M	13	5,4	5,2	1,04	0,96	STRAIGHT
11	M	14	9	8,4	1,07	0,93	STRAIGHT
12	F	13	8,4	8,1	1,04	0,96	STRAIGHT
13	F	14	10,4	10,3	1,01	0,99	STRAIGHT
14	F	15	9,8	10,3	0,95	1,05	STRAIGHT
15	F	10	3,6	3,7	0,97	1,03	STRAIGHT
16	M	17	6,1	6	1,02	0,98	STRAIGHT
17	F	14	10,8	10,7	1,01	0,99	STRAIGHT
18	F	14	5,1	4,9	1,04	0,96	STRAIGHT
19	M	10	7,8	7,5	1,04	0,96	STRAIGHT
20	M	8	3,3	3,5	0,94	1,06	STRAIGHT
21	F	14	7,9	8,2	0,96	1,04	STRAIGHT
22	F	14	11,1	10,7	1,04	0,96	STRAIGHT
23	F	14	7,4	6,7	1,10	0,91	STRAIGHT
24	M	15	10,1	10,2	0,99	1,01	STRAIGHT
25	F	11	5,1	5,1	1,00	1,00	STRAIGHT
26	M	14	8,4	8,1	1,04	0,96	STRAIGHT
27	M	13	6,1	5,8	1,05	0,95	STRAIGHT
28	F	12	4,7	4,8	0,98	1,02	STRAIGHT

There was no pelvic tilt in both groups.

### The measurements

**Fig. 1:**
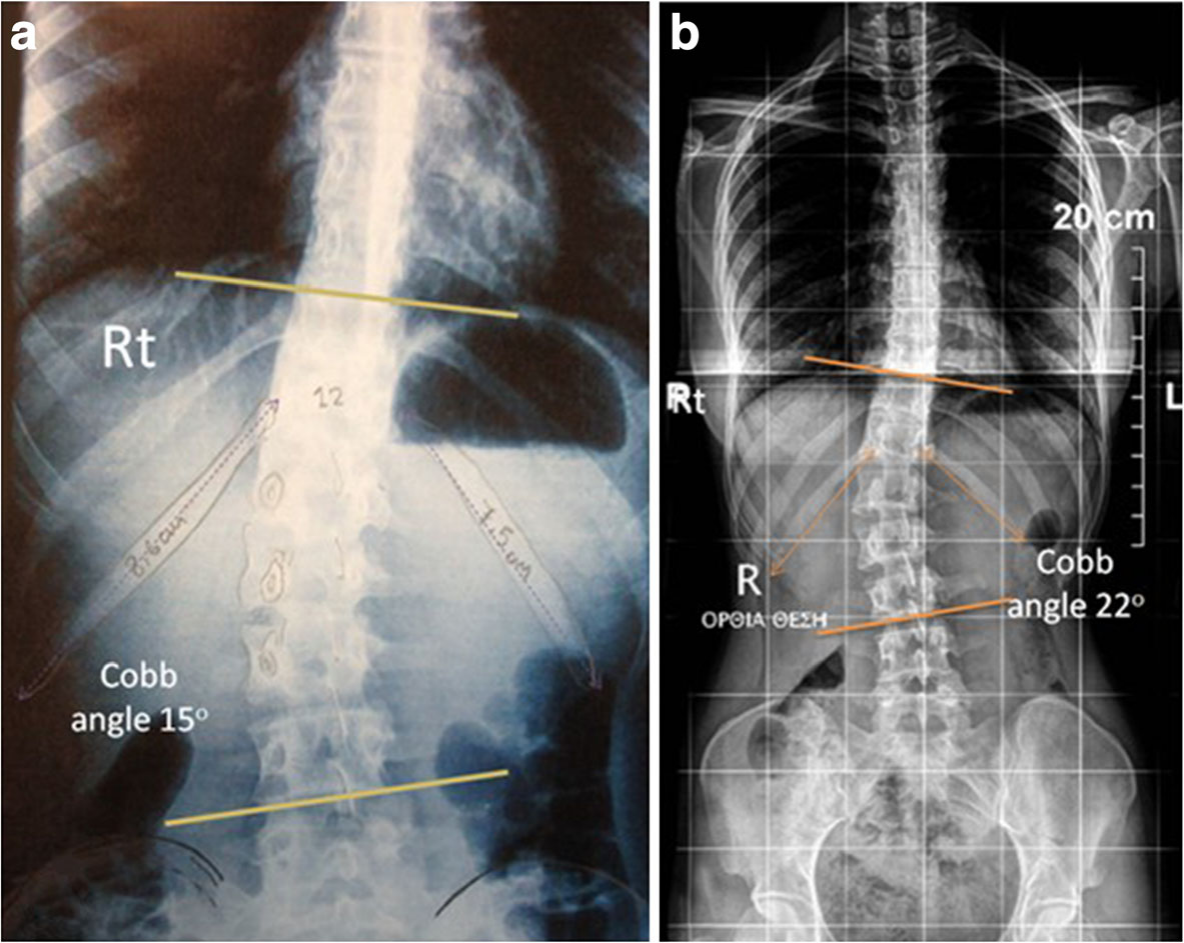
**a**. Right lumbar idiopathic curve 15° of Cobb angle, the right 12th rib is longer, similarly in figure **b**, in a right lumbar idiopathic curve 22° of Cobb angle, the right 12th rib is longer

**Fig. 2:**
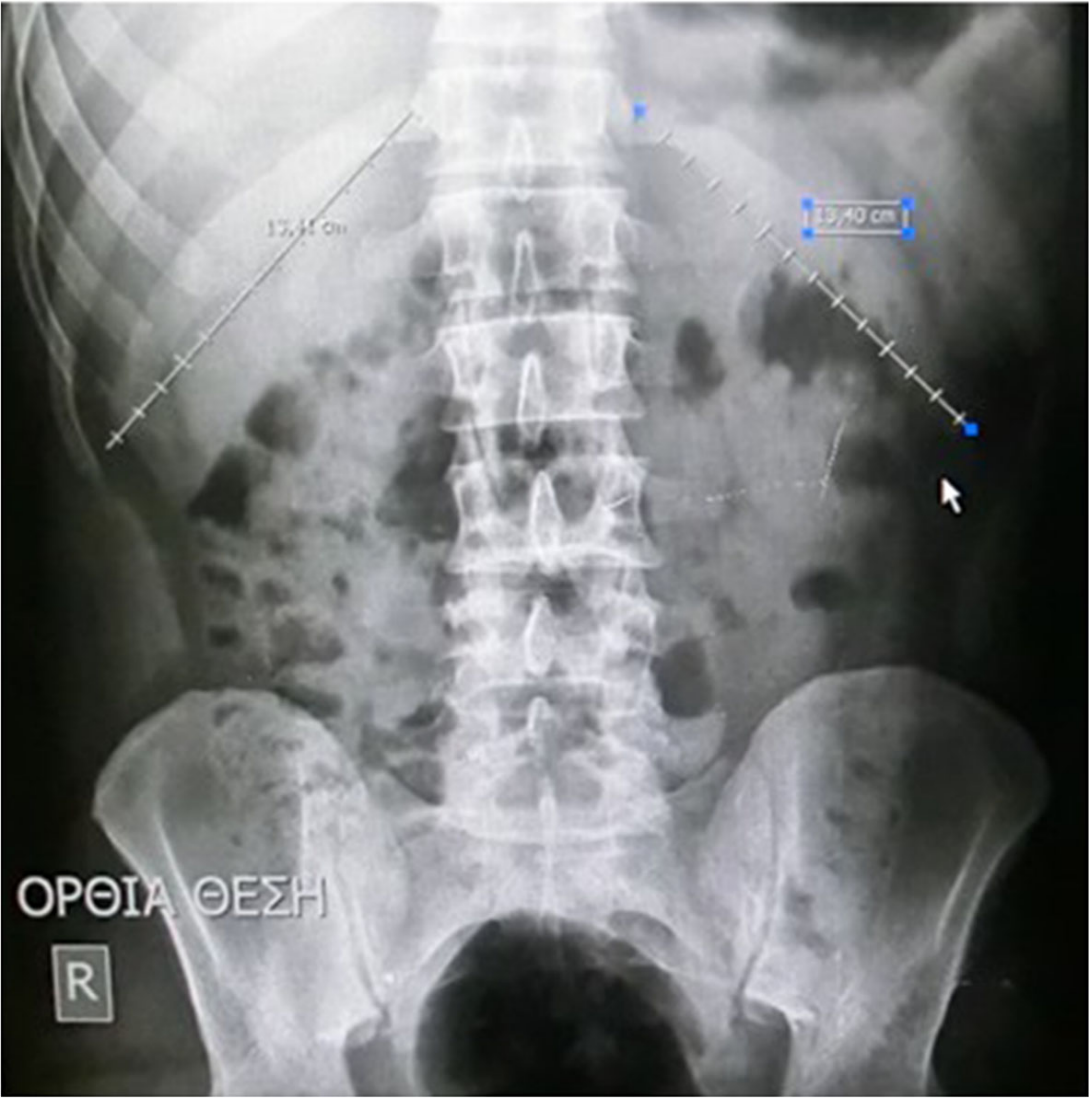
In this straight spine, the right 12th rib measures 13,41 cm while the left 13,40 cm respectively

The length in cm from its head to the lateral end point of each of the right and the left 12th rib was measured in the two groups of examinees’ P-A spinal radiographs, Figs. 1 and 2. The ratio of the right/left 12th rib lengths and its reliability calculated. We also assess the existence of vertebral rotation, observing the symmetry of the pedicles. If 12th vertebra is rotated then the curve could be thoracolumbar.

### The statistical analysis

The difference of the ratio between the two groups was tested (Mann-Whitney) and the correlation between the ratio and the Cobb angle estimated (Spearman’s rho). The statistical analysis was done using the SPSS package v22.

### Study design

This is a case-control study and of level of evidence III.

## Results

The ratio reliability study showed intra-observer +/−0,036 and the inter-observer error +/−0,042 respectively in terms of 95 % confidence limit of the error of measurements.

The 12th rib was longer on the side of the curve convexity in 12 children with LLC and equal in two patients with lumbar scoliosis.

The 12th rib ratios of the children with lumbar curves were statistically significantly greater than in those with straight spines (Mann-Whitney U = 71,000, *p* < 0,001).

**Fig. 3:**
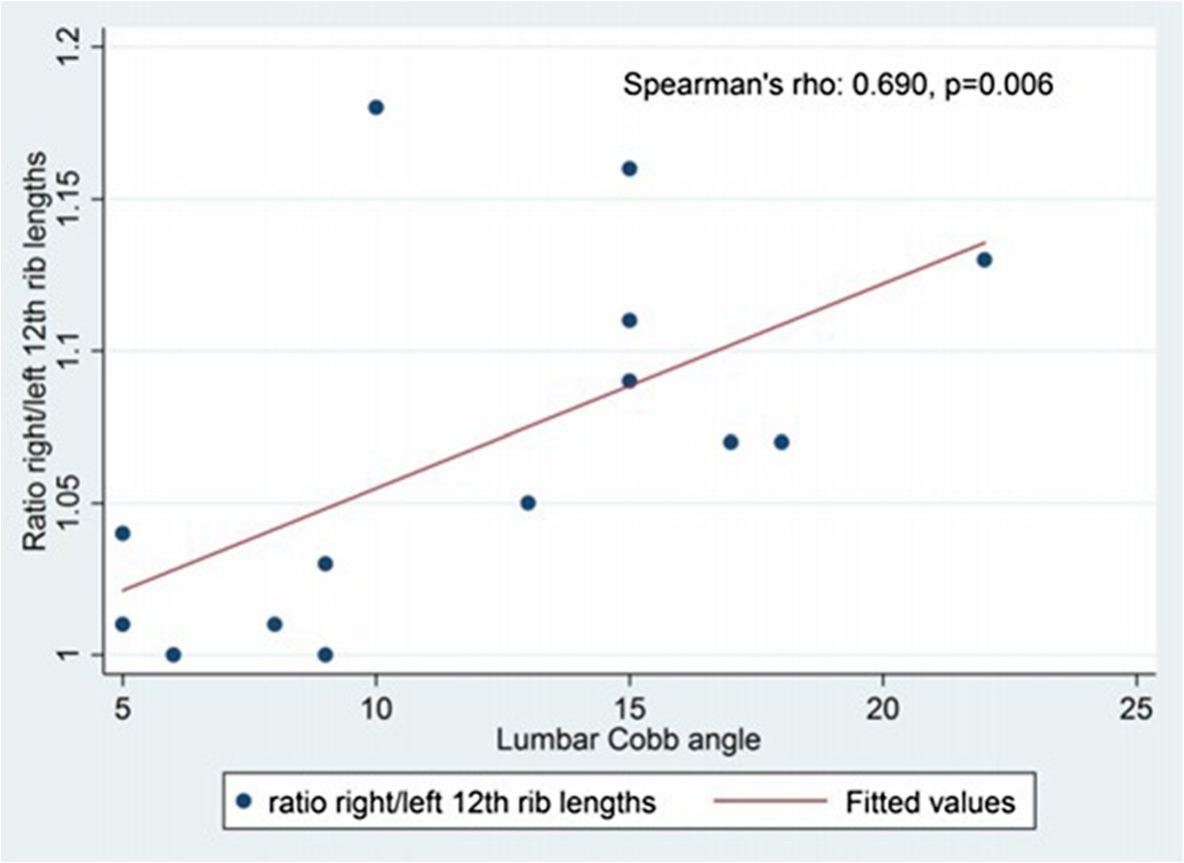
Scatter plot of lumbar Cobb angle by ratio right/left rib lengths. The correlation was statistically significant, (*r* = 0,690, *p* = 0,006)

The correlation of the twelfth rib ratio with Cobb angle was statistically significant, (Spearman’s rho = 0,690, *p* = 0,006), Fig. 3.

The 12th thoracic vertebrae show no axial rotation (or minimal) in the LLC and no rotation on straight spine groups.

## Discussion and conclusions

It is not possible, at present, to determine whether the 12th convex rib lengthening is congenitally lengthened, induced mechanically, or both. One question could be posed on whether the asymmetry in the rib length preceded the curves (possibly causal) or developed as a reaction to the increased demand. Answering this question it is stated that our data are cross-sectional, so we can only comment on data we do have (the radiographs obtained at a certain age). We do not have longitudinal data to address this question.

**Fig. 4:**
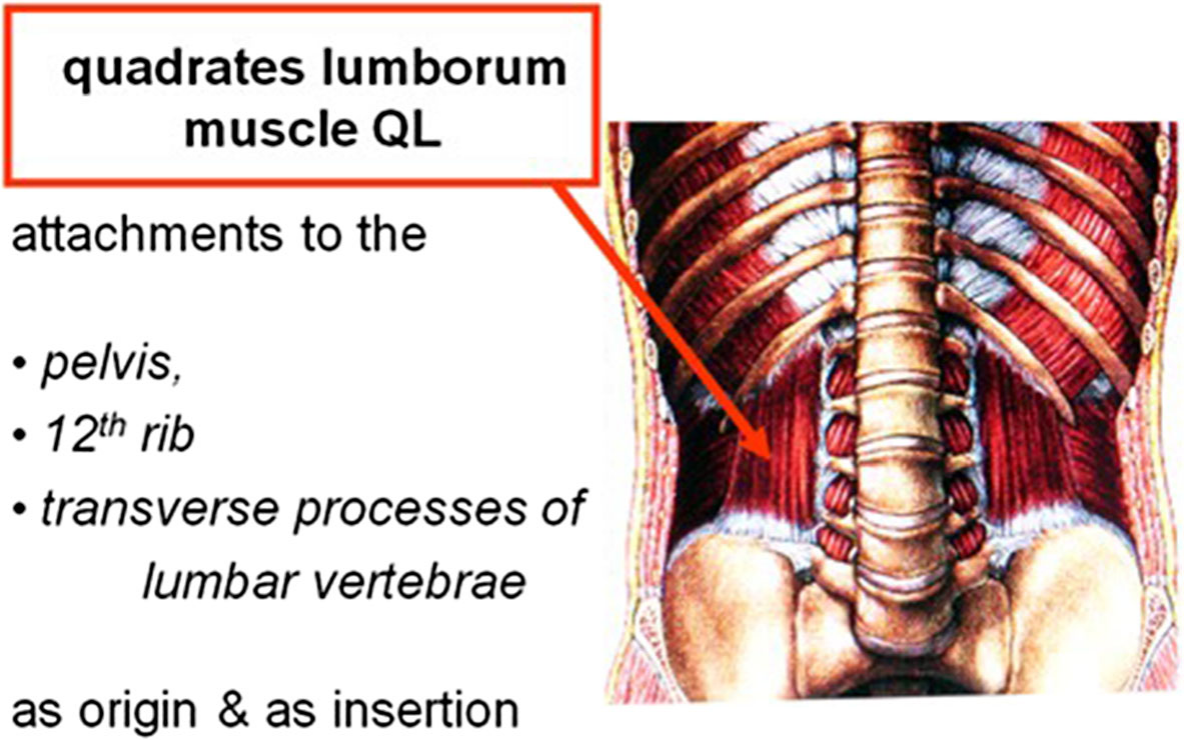
Quadrates lumborum attachments

Several small muscles are attached to the 12th ribs. Attachments to the 12th ribs include diaphragm, quadrates lumborum, Fig. 4, internal and external intercostals, serratus posterior inferior, short and long rib elevators, external oblique abdominal, internal oblique abdominal, transversus abdominis, iliocostalis and longissimus thoracis.

We focus attention here on the largest of these muscles namely, QL. The largest muscles, attached to the 12th ribs namely quadrates lumborum, are likely to exert the greatest forces on the 12th ribs.

QL has attachments to the pelvis, 12th ribs and transverse processes of lumbar vertebrae as origins and as insertions.

**Fig. 5:**
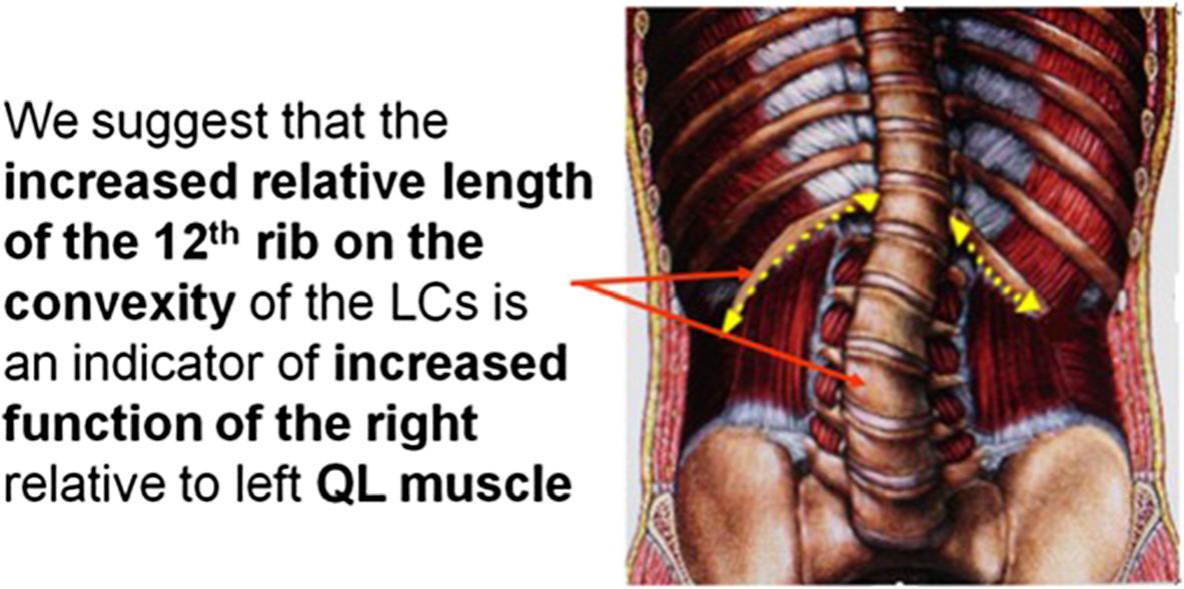
Our hypothesis on the increased relative length of the 12th rib on the convexity of right lumbar idiopathic scoliotic curves

Riddle and Roaf [2], using electromyography in paralytic and idiopathic thoracic and paralytic lumbar scoliosis, reported the spinal muscles were significantly stronger on the convex side at the apex of the curve. Given the increased muscle activity on the lumbar curve convexity and similar to the interpretations of earlier workers outlined above in the Background section, we suggest two hypotheses, relatively increased activity of the right QL muscle causes the LLCs (first hypothesis); or counteracts the lumbar curvature as part of the body’s attempt to compensate for the curvature (second hypothesis). These hypotheses may be tested by electrical stimulation studies of QL muscles in subjects with lumbar IS by revealing respectively curve worsening or correction. We suggest that one mechanism leading to relatively increased length of the right 12 ribs is mechanotransduction [9] in accordance with Wolff’s and Pauwels Laws, Fig. 5.

**Wolff’s law** is a theory developed by the German anatomist and surgeon Julius Wolff (1836–1902) in the 19th century that states that bone in a healthy person or animal will adapt to the loads under which it is placed. If loading on a particular bone increases, the bone will remodel itself over time to become stronger to resist that sort of loading, [10, 11].

**Pauwels’ law** states that intermittent pressure within the physiological limits of stress and strain stimulates the growth plates of a healthy bone, [12, 13].

The concept of this short paper was in brief published [14].

## Abbreviations

IS: Idiopathic scoliosis
QL: Quadrates lumborum
UPD: Outpatient department
LLC: Lateral lumbar curves
SS: Statistically significantly
